# Inducible Control of Subcellular RNA Localization Using a Synthetic Protein-RNA Aptamer Interaction

**DOI:** 10.1371/journal.pone.0046868

**Published:** 2012-10-08

**Authors:** Brian J. Belmont, Jacquin C. Niles

**Affiliations:** Department of Biological Engineering, Massachusetts Institute of Technology, Cambridge, Massachusetts, United States of America; Duke University Medical Center, United States of America

## Abstract

Evidence is accumulating in support of the functional importance of subcellular RNA localization in diverse biological contexts. In different cell types, distinct RNA localization patterns are frequently observed, and the available data indicate that this is achieved through a series of highly coordinated events. Classically, *cis*–elements within the RNA to be localized are recognized by RNA-binding proteins (RBPs), which then direct specific localization of a target RNA. Until now, the precise control of the spatiotemporal parameters inherent to regulating RNA localization has not been experimentally possible. Here, we demonstrate the development and use of a chemically–inducible RNA–protein interaction to regulate subcellular RNA localization. Our system is composed primarily of two parts: (i) the Tet Repressor protein (TetR) genetically fused to proteins natively involved in localizing endogenous transcripts; and (ii) a target transcript containing genetically encoded TetR–binding RNA aptamers. TetR–fusion protein binding to the target RNA and subsequent localization of the latter are directly regulated by doxycycline. Using this platform, we demonstrate that enhanced and controlled subcellular localization of engineered transcripts are achievable. We also analyze rules for forward engineering this RNA localization system in an effort to facilitate its straightforward application to studying RNA localization more generally.

## Introduction

**Figure 1:**
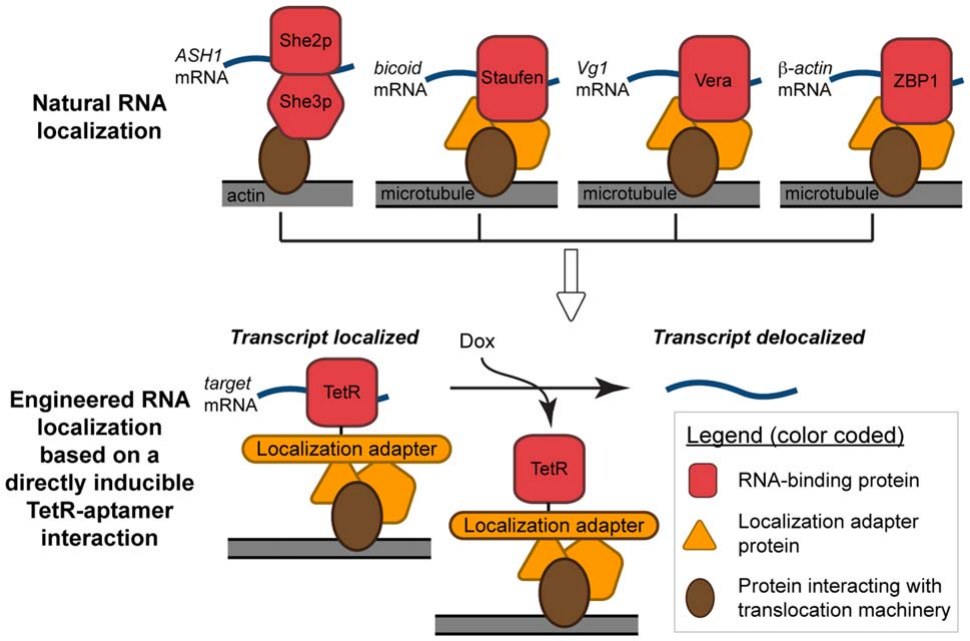
Overview of several examples of natural transcript localization schemes. Shown (left to right): bud localization of *ASH1* mRNA in *Saccharomyces cerevisiae*; polarization of *bicoid* mRNA in *Drosophila melanogaster* embryos; vegetal cortex enrichment of *Vg1* mRNA in *Xenopus laevis* oocytes; and filopodial enrichment of *ß*–*actin* mRNA in migrating fibroblasts. These systems define the core principles inspiring the design of the depicted TetR–aptamer system for achieving directly regulated transcript localization.

Specific subcellular RNA localization has long been recognized as a central mechanism regulating important biological processes, such as mating type switching in *Saccharomyces cerevisiae*, defining body axis polarity in *Drosophila* and *C. elegans*, fibroblast and neuronal growth cone migration, synaptic plasticity, and storage of maternally–derived transcripts [for some pertinent reviews, see: [1], [2], [3]]. Several recent studies examining genome–wide cellular RNA distribution have provided a new appreciation of the pervasiveness of transcript–specific localization into distinctive subcellular patterns [4], [5], [6], [7]. With the exception of a few prominent examples, however, the functional significance of such extensive, region–specific RNA localization remains largely unknown. Nevertheless, some important molecular details have emerged on how RNA localization is achieved and regulated. A recurrent theme is the presence of one or more *cis*–elements within localized transcripts that are recognized by cognate RNA-binding proteins (RBPs) (Fig. 1). Through protein–protein interactions scaffolded by the RBP, these ribonucleoprotein (RNP) complexes can be actively transported to or become entrapped within specific subcellular regions [3]. Depending on the region to which mRNAs are targeted, they can remain translationally silent until their encoded proteins are required [8], [9], [10]. Alternatively, transcripts can immediately become translationally active, giving rise to localized protein synthesis [11], [12].

In the relatively few instances where the functional importance of subcellular RNA localization is validated, it is clear this is a dynamic and highly regulated process. Regulation can occur at three main points: Step I–the interaction of *cis*–RNA elements with the cognate RBPs; Step II–assembly, transport and targeting of the RNP complex to the appropriate location; and Step III–translational de–repression at the site of RNA localization. Modulation of the RNA-protein interaction during Steps I and III is understood in some detail, and may be achieved, for example, via transcription–mediated changes [13] or post–translational modification [11], [12] that alter the concentration or binding properties of the RBP. Typically, complex and poorly understood signaling processes that cannot be precisely manipulated experimentally trigger these regulatory events [14], [15]. Step II involves a complex and dynamic interplay between many accessory proteins. Therefore, engineering regulation at Steps I and III is, in principle, more readily attainable.

Dissecting the functional significance of subcellular transcript localization presents a major but important challenge. In much the same way that systems for inducible and dynamic regulation of gene expression have been invaluable in establishing the roles proteins play in various biological processes, we envision that the ability to modulate subcellular RNA localization will play an indispensable role in establishing its functional significance more globally. Simultaneously, as we gain a better understanding of how RNA localization shapes cellular function, opportunities to use this knowledge in design–oriented applications in areas such as synthetic biology and neurobiology will emerge. Towards this long–term objective, previous efforts have used endogenously recognized *cis*–elements introduced into target transcripts that are localized exclusively by native mechanisms [16], [17], [18]. A significant drawback to this approach, however, is that there is no straightforward way to precisely control localization in a manner orthogonal to normal cellular physiology. Alternatively, heterologous *cis*–elements based on MS2 and boxB RNA binding sites have been encoded into target transcripts and co–expressed with protein fusions between the MS2 coat or λN proteins and endogenous localization effector proteins [19], [20]. This approach can permit regulation of transcript localization by using inducible promoters to control synthesis of the RBP–effector protein fusion [21]. However, an important limitation of this strategy is its inability to recapitulate some functionally critical aspects of endogenous localization control, namely, the direct reversibility of the RBP–target transcript interaction [11], and the highly dynamic control attainable by eliminating the need for new transcription and/or regulatory protein synthesis or turnover [11], [22], [23].

Here, we present a general strategy that overcomes the inherent limitations above. Guided by insights from several natural localization mechanisms [11], [12], we focus on regulating localization by directly controlling whether a target transcript can engage the RNA localization machinery (Steps I and III). This is achieved by genetically encoding tetracycline–inducible, TetR–binding aptamers [24], [25] in the target transcript. We reason that the biochemical information necessary for generating localization–competent RNPs is encoded within the RBP and/or other effector proteins within the RNP complex natively involved in localization. Thus, we use TetR–RBP/localization effector protein fusions to bridge a target transcript with the cell’s localization machinery. In this scheme, when the TetR fusion protein engages a transcript (doxycycline absent) it is effectively localized. However, localization is disrupted by directly inducing disengagement of the target transcript (doxycycline present) from the foundational TetR–RNA interaction scaffolding formation of the localization–competent RNP complex. We have used the model of asymmetric RNA localization in *S. cerevisiae* [1], [26], [27] to establish our design principles. We demonstrate that TetR tolerates fusion to multiple proteins involved in RNA localization with no apparent defect in binding to its RNA aptamer and inducibility by a tetracycline analog. These TetR fusion proteins also effectively direct specific and inducible subcellular localization of a reporter transcript. Additionally, we have defined rules governing the TetR–binding capacity of the aptamers within the target transcript. Overall, due to the inherent flexibility of this system, we envision that it can serve as a platform for both recapitulating and creating more complex and functionally relevant RNA localization schemes in a variety of organisms.

## Results and Discussion

*Saccharomyces cerevisiae* was used as a model context for this work, as many of the molecular details underlying its natural RNA localization machinery have been elucidated. Several transcripts, such as the *ASH1* mRNA, are asymmetrically trafficked to the growing bud in *S. cerevisiae* [reviewed in [1], [26], [27]]. Target transcripts frequently contain unique sequence elements that are directly recognized by the RBP, She2p, in conjunction with She3p [28]. This complex interacts with the myosin motor protein, Myo4p, which directs the protein–mRNA complex using the cellular actin network to the distal portion of the growing bud or neck of a mating projection [29].

### Generating TetR–fusions and Evaluating their Ability to Localize Target Transcripts

**Figure 2:**
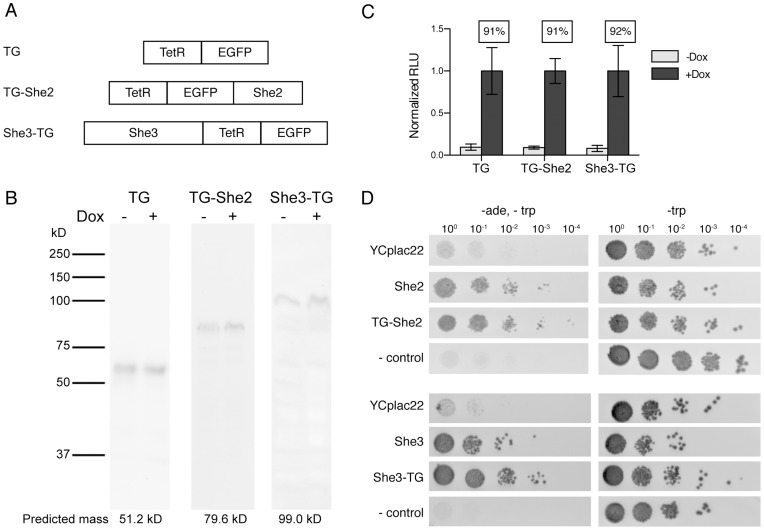
The TetR component in all fusion proteins remains functional. (A) Schematic of the TetR fusions to EGFP and full-length She2p and She3p used in this study. (B) Western blotting using an anti-TetR antibody indicated that TG, TG–She2 and She3–TG are all produced as intact, soluble proteins. The predicted molecular weight for each is noted. (C) The TetR component in all fusion proteins remains functional as determined by their ability to repress firefly luciferase expression from a luciferase transcript containing a TetR aptamer within its *5′UTR*. Doxycycline (Dox) relieves this effect as expected. The percent repression (ratio of – Dox to + Dox luciferase signal) is indicated above each bar. Error bars indicate 95% confidence intervals. (*D*) Yeast genetic assay for asymmetric *ASH1* mRNA localization. Ten-fold dilutions of the K5547 (to test She2 and TG–She2 function) or K4822 (to test She3 and She3–TG function) yeast strains carrying the indicated plasmid were plated on either – tryptophan or – tryptophan/− adenine plates, and grown for three days. The control strain is an *ADE2* mutant.

The components needed to achieve inducible transcript subcellular localization are summarized in Figure 1. In this work, reporter transcripts targeted for subcellular localization encoded a non–fluorescent Venus yellow fluorescent protein variant (vYFPΔ) [30]. The TetR–binding aptamer, **5–1.2**, was genetically encoded within the *5′* or *3′ UTR* of this transcript as either a single aptamer (*5′* and *3′ UTR*) or in tandem arrays (*3′UTR* only). Two synthetic RBPs were designed and consisted of a TetR–EGFP (TG) core fused either to the N–terminus of full–length She2p to give TG–She2 or the C–terminus of full–length She3p to give She3–TG (Fig. 2A). The EGFP component facilitated direct visualization of the subcellular location of TG–She2 and She3–TG by fluorescence microscopy imaging.

We first established that all three fusions are produced as soluble, full–length proteins by Western blotting analysis (Figure 2B). Next, we demonstrated that the TetR, She2p, and She3p components all retained their expected functionality within TG, TG–She2 and She3–TG. We determined functionality of the TetR component by quantifying how effectively TG, TG–She2 and She3–TG inducibly controlled translation of a firefly luciferase reporter transcript containing a **5–1.2** aptamer within its *5′UTR*, as described previously [25]. All TetR fusion proteins repressed translation by ∼90% in the absence of a TetR inducer, and this effect was reversed by doxycycline (Figure 2C). This data confirmed that TetR tolerates both C– and N– terminal fusion to endogenous proteins essential to RNA localization with retention of its expected doxycycline–inducible RNA binding properties.

Next, we tested the functionality of She2p and She3p within TG–She2 and She3–TG using a plate–based growth assay in which effective asymmetric distribution of Ash1p is made dependent on functional She2p and She3p within TG–She2 and She3–TG. Asymmetric Ash1p accumulation within the daughter cell (functional She2p or She3p required) allows the maternal *HO* promoter to remain active to drive expression of Ade2p, and promote growth on – ade media. However, Ash1p symmetrically distributed between daughter and mother cells (She2p or She3p disrupted) represses the *HO* promoter in both, leading to overall growth suppression on – ade media [25], [31]. Therefore, by expressing TG–She2 and She3–TG in *she2*Δ or *she3*Δ yeast strains, respectively, we expect to observe growth on – ade media only if the She2p and She3p components remain functional. As shown in Fig. 2D, this is indeed the case. Altogether, these data confirm modular functionality of each component within both TG–She2 and She3–TG, and their suitability for regulating subcellular RNA localization.

### Transcript Localization using a Single TetR Aptamer within its *5′UTR*

**Figure 3:**
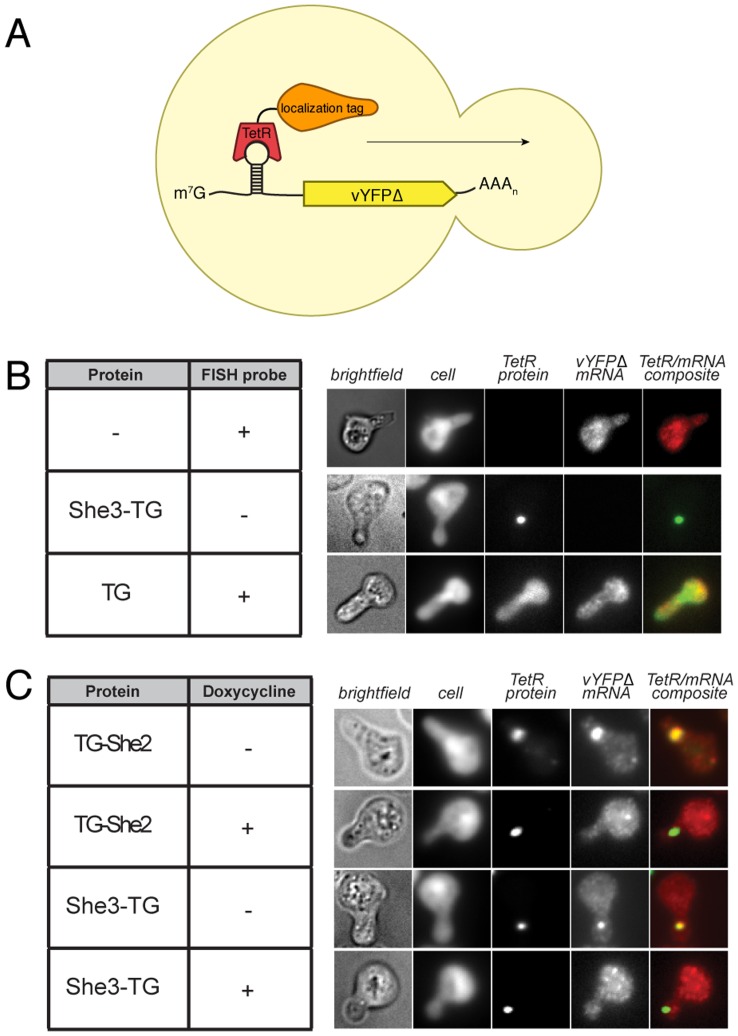
Fluorescence imaging data show that the 5–1.2–vYFPΔ transcript with a single TetR aptamer within its *5′UTR* can be effectively localized in a doxycycline–dependent manner. (A) A schematic of the **5–1.2**–vYFPΔ transcript, which encodes a non–fluorescent Venus YFP (vYFP), is shown. Cells were arrested in G1–phase prior to imaging. The cell body (Whole Cell Stain), TetR fusion (direct EGFP fluorescence) and **5–1.2**–vYFPΔ (FISH using Cy5–labeled probes) are visualized in cells expressing TG, TG–She2, She3–TG or neither. These data establish: (i) the absence of crosstalk between the different spectral channels; (ii) that TG alone does not localize and cannot localize the **5–1.2**–vYFPΔ transcript; and (iii) the **5–1.2**–vYFPΔ transcript is not localized at baseline. (B) Images are as in (A), but doxycycline is either absent (transcript localized) or present (transcript delocalized).

We first tested localization of the **5–1.2**–vYFPΔ transcript containing a single aptamer within its *5′UTR*. Our previous work established that when presented in different *5′UTR* sequence contexts, this aptamer interacts with TetR to robustly regulate translation [25]. Furthermore, accumulating evidence indicates that maintaining RNA in a translationally repressed state is critical for its efficient transport [32]. Therefore, we reasoned that with a *5′UTR* aptamer, translational repression synergistic with efficient localization could be simultaneously achieved. To evaluate attaining small molecule–regulated control over subcellular localization, *she2*Δ or *she3*Δ yeast were co-transformed with TG–She2 or She3–TG, respectively, and the **5–1.2**–vYFPΔ reporter transcript. Initially, we encoded the TetR–binding aptamer within the 5*′UTR* sequence context used in the translation repression assays (Fig. 3A). The spatial distribution of TG–She2/She3–TG and the **5–1.2**–vYFPΔ transcript were established by the EGFP signal, and fluorescence *in situ* hybridization (FISH) with Cy5–labeled probes, respectively. To minimize experimenter–introduced bias in our analyses of subcellular localization, we used computer–aided methods (see Methods). Pilot experiments indicated, however, that conjoined mother–daughter pairs were difficult to distinguish from clumped cells. To circumvent this problem, we arrested cells in G1–phase by adding mating factor. This prevents complete budding, and induces formation of mating projections, or shmoos, which are single cells readily distinguished from cell clumps. Previous work has established that this manipulation does not disrupt the asymmetric RNA localization machinery [33]. Overall, we were able to implement a robust and higher throughput computer–aided pipeline for image analysis of subcellular localization using G1–phase arrested cells.

To ensure that crosstalk between the EGFP and Cy5 fluorescence channels would not complicate analysis, we visualized the Cy5 channel after performing FISH in cells not expressing a TetR–fusion protein, and EGFP in She3–TG expressing cells without the addition of Cy5–labeled FISH probes (Fig. 3B). No detectable crosstalk was observed between the two fluorescence channels. We visualized cells co–expressing the **5–1.2**–vYFPΔ transcript and either TG–She2 or She3–TG (Fig. 3C). Both strains showed an intense signal of concentrated TetR–fusion protein in the shmoo neck, and the co–localizing transcript signal appeared greatly enhanced (Fig. 3C). This co–localization suggests that TG–She2 and She3–TG are recruiting the reporter transcript to a defined subcellular location. In this model, doxycycline is expected to disrupt the interaction between the TetR–fusion protein and **5–1.2**–vYFPΔ transcript. This should abrogate enhanced co–localization of the transcript with TG–She2 or She3–TG without altering localization of either fusion protein. This is indeed the observation when cells were grown in the presence of doxycycline, indicating that the TetR–aptamer interaction is mediating the observed transcript localization (Fig. 3C).

**Figure 4:**
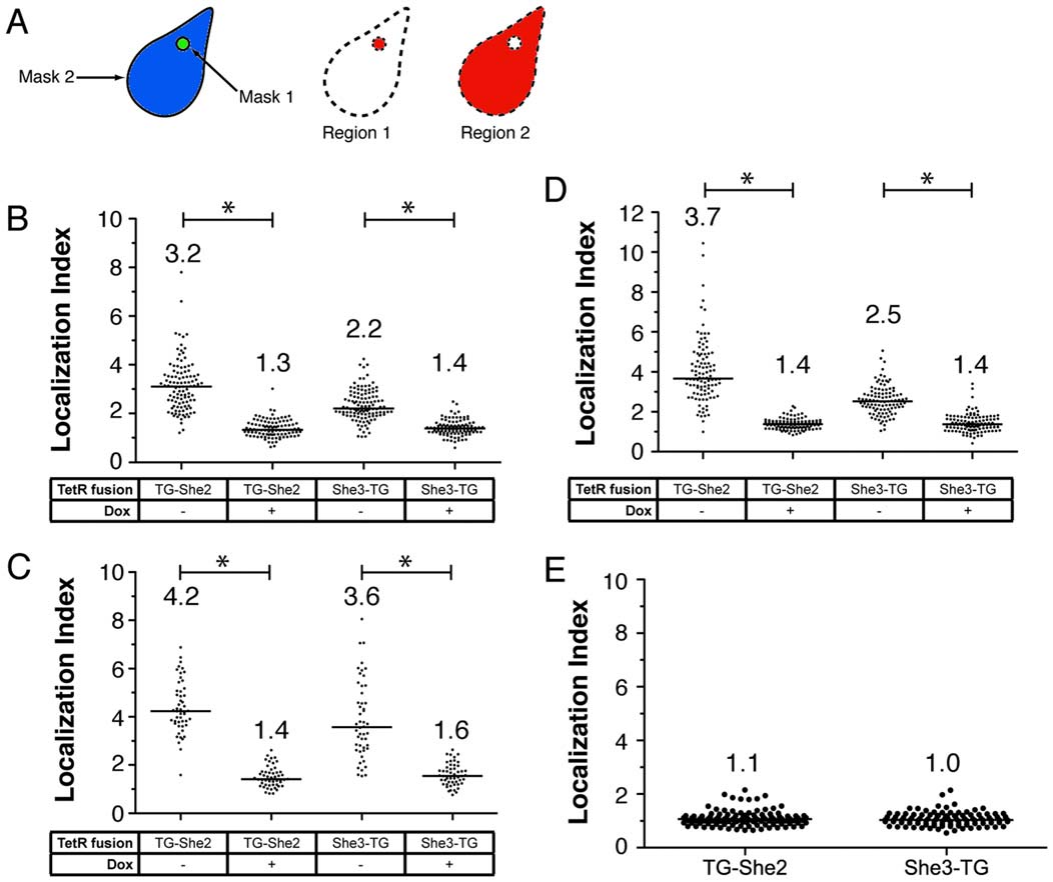
Quantitation of the localization attainable using a single TetR aptamer located within the *5′UTR* of a reporter transcript. (A) Schematic of yeast image processing used in assigning Localization Indexes. Mask 1 defines the She2–TG or She3–TG protein foci. Mask 2 defines the entire yeast cell. Region 1 is the area inside Mask1 and Region 2 is the area outside of Mask1 but inside of Mask 2. (B) Computer-aided Localization Index measurements determined for yeast expressing **5–1.2**–vYFPΔ and TG–She2 or She3–TG. Cells were grown in the absence or presence of doxycycline. Each dot represents single cell measurements. At least 100 cells were counted for each condition. (C) Manually–determined Localization Index measurements for the same images used in (B). At least 50 cells were analyzed for each condition. (D) Localization measurements determined as in (B), but in cells where the *ADH1* promoter drives production of the **5–1.2**–vYFPΔ reporter transcript. (E) Analysis of cells expressing the **No aptamer**–vYFPΔ transcript. In all cases, the line and numbers above each data set represent the median Localization Index for that condition. A bar with an asterisk (*) above denotes a statistically significant difference (*p*<0.0001), as determined by a two-tailed *t*–test, between the indicated conditions.

For a more quantitative assessment of the RNA localization capabilities of our system and to facilitate more standardized comparisons under varying experimental conditions, we devised a straightforward analytical procedure to determine TetR–fusion protein and target RNA co–localization. After capturing microscopy images, a computational algorithm was used to define two masks, one defining the TG–She2/She3–TG foci (Mask 1) and another the entire cell body (Mask 2) (Fig. 4A). Two regions were defined based on these masks, namely: *(i)* Region 1, which is the area encompassed by Mask 1, and thus defines the region within which enhanced target transcript accumulation occurs; and Region 2, which is the area encompassed by Mask 2, but excludes Region 1. We defined a Localization Index as the ratio of the mean Cy5 fluorescence signal density of Region 1 to that of Region 2. Effectively, this metric reflects the ratio of the average co–localized to non–localized reporter transcript signal.

We applied this analysis method to cells co–expressing the **5–1.2**–vYFPΔ transcript and either TG–She2 or She3–TG, in either the presence or absence of doxycycline (Fig. 4B). Cells with either protein fusion show a significantly increased Localization Index. This effect is abolished by doxycycline. Interestingly, strains expressing TG–She2 achieve higher transcript localization when compared to strains expressing She3–TG, which may suggest that co–opting effector proteins most upstream in the RNA localization pathway may be an important strategy for maximizing overall localization efficiency using our regulated, synthetic system. To ensure that the computational image analysis method accurately represented the localization phenotype, manual masking of the same images and subsequent calculations were performed (Fig. 4C). The data from each analysis method correlates well, although manual masking appears to result in a more profound localization effect. This is likely due to the higher stringency with which mask boundaries could be assigned manually. Since both methods yielded qualitatively similar results, and due to the rapid throughput of the computer–aided approach, we performed all subsequent analyses computationally.

In the previous experiment, the strong *TEF1* promoter was used to produce the **5–1.2**–vYFPΔ reporter transcript at high levels [34]. We sought to determine if lowered expression of the reporter mRNA would enhance the localization dynamic range of our system, perhaps due to a higher ratio of TetR–fusion protein to target transcript. We replaced the *TEF1* promoter with a lower activity *ADH1* promoter [34], and performed localization analysis. Indeed, this led to significantly higher localization indices in the uninduced state when either the TG-She2 (*p*<0.0001) or She3-TG (*p* = 0.01) protein was expressed, without negatively impacting doxycycline–induced reversibility of localization (Fig. 4D). These data indicate that titrating the relative expression level of the localization system components is an important parameter when fine–tuning this system to optimize functionality for a given application. However, for all subsequent analysis, we continued to use the *TEF1* promoter for reporter mRNA expression.

In each of the above cases, inducing the TetR–aptamer interaction with doxycycline substantially abrogated the transcript localization phenotype, but full induction (Localization Index = 1) is not observed. This may be due to residual interaction between the TetR aptamer and TG–She2/She3–TG even in the presence of doxycycline that causes a slight enhancement in transcript signal co–localizing with the TetR fusion proteins, or bias introduced by the analysis method. To explore the cause of the apparent incomplete induction, we performed localization analysis with strains co–expressing TG–She2/She3–TG and the **No aptamer**–vYFPΔ transcript, which lacked a TetR–binding aptamer. The localization indices observed with **No aptamer**–vYFPΔ were closer to one than those for **5–1.2**_vYFPΔ (Fig. 4E). These data suggest that some residual affinity between the **5–1.2** aptamer and TetR remains in the presence of doxycycline, and that the analysis method is capable of discerning slight differences between strains and induction conditions.

### Transcript Localization with a Single TetR Aptamer within the *3′UTR*

As shown above, effective and inducible RNA localization is attainable with a single aptamer positioned within a target transcript’s *5′UTR*. However, many naturally occurring instances of RNA localization are mediated by *cis*–elements located with the *3′UTR* of the target transcript [3]. Therefore, we sought to address whether our TetR–aptamer system could be used to achieve transcript localization in a manner most closely recapitulating this common endogenous mechanism. To synthetically recapitulate this, but with precise exogenous chemical control, we systematically defined the requirements for achieving inducible RNA localization using *3′UTR*–encoded TetR aptamers. Along with defining a functional localization system having experimental utility, we examined failed designs to better understand the link between preserved protein–RNA interaction biochemistry and observable RNA localization. We believe this is useful for two main reasons. First, if poor transcript localization efficiency were due to sub–optimal aptamer display preventing high affinity interaction with TG–She2 and She3–TG, this would allow us to emphasize pursuing design–oriented and/or selection–based strategies to define an aptamer display context compatible with high affinity interaction with the TetR fusion proteins. Second, our previous efforts regulating translation using TetR–aptamer interactions suggested that an intact protein–RNA interaction is necessary but not sufficient for functionality [24], [25], and so we wanted to understand whether this is similarly true for RNA localization.

**Figure 5:**
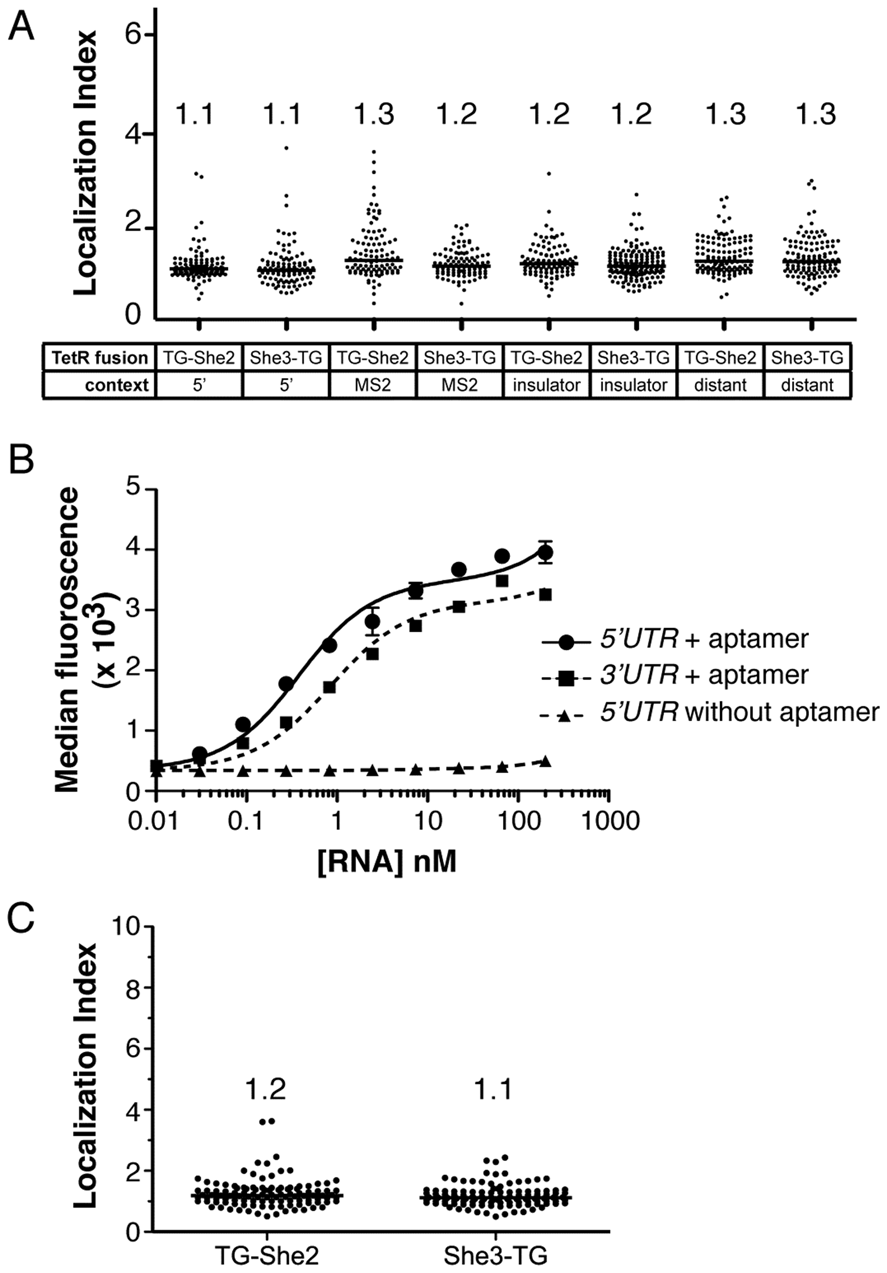
A single 3′UTR–encoded TetR aptamer interacts with TetR *in vitro*, but does not mediate significant transcript localization *in vivo*. (A) Localization indexes determined upon co–expressing the vYFPΔ–**5–1.2** transcript with either TG–She2 or She3–TG. Measurements are reported for four different sequence contexts within which **5–1.2** was presented in the *3′UTR*. (B) Binding data for the interaction of TetR with *in vitro* transcribed RNA identical to either the aptamer containing regions of **5–1.2**–vYFPΔ (1–180 bases="*5′UTR* + aptamer”) or vYFPΔ–**5–1.2 (**613–946 bases="*3′UTR* + aptamer”), and the **No Aptamer**–vYFPΔ (1–135 bases = *5′UTR* without aptamer). (C) Localization Indexes for the vYFPΔ–**5–1.2 (** *5′UTR* context**)** transcript with a stem structure included within the *5′UTR*. The numbers above each data set are the median Localization Index for that condition.

We began by encoding a single **5–1.2** aptamer within the *3′UTR* of our vYFPΔ reporter (vYFPΔ_**5–1.2**), and co–expressing this transcript with either TG–She2 or She3–TG and measuring vYFPΔ_**5–1.2** localization as before (Fig. 5A). We hypothesized that the sequence context within which the TetR aptamer is presented could potentially impact its ability to productively interact with TG–She2 and/or She3–TG. Therefore, four vYFPΔ_**5–1.2** constructs were tested, each differing only in the sequence context immediately flanking the aptamer (see Table 1 for sequence information). The contexts tested were: (i) the same as that successfully used in the **5–1.2**_vYFPΔ construct; (ii) the same as was successfully used previously with MS2–binding aptamers in the *3′UTR* [35]; (iii) a flanking insulator sequence predicted to have minimal secondary structure [36], and that could facilitate modular folding and presentation of the TetR aptamer; and (iv) a 73 base insertion between the vYFPΔ stop codon and the aptamer. This increased the distance between the stop codon and aptamer beyond that used in (i)–(iii), and provided an opportunity to understand how this variable impacted localization efficiency. In all four aptamer contexts, no enhanced vYFPΔ_**5–1.2** localization was observed using either TG–She2 or She3–TG (Fig. 5B).

**Table 1:** Aptamer and UTR sequences.

Aptamer sequence context	Sequence
**5–1.2 aptamer**	GGATCCAGGCAGAGAAAGGTCGATACGGACGGAATGTGATGGCCTGGATCC
***5′UTR***	AATTATCTA–(**5–1.2**)–AACACAAAACTCGAGAACAT*ATG*
***5′UTR*** **, “5** ***′*** ** context”**	**TAA**TCTAGAAATTATCTA–(**5–1.2**)–AACACAAAACTCGAGAACATCCCGGGAAAA TCTAGAATTCCTT
***3′UTR*** **, “MS2 context”**	**TAA**TCTAGAGATCCTAAGGTACCTAATTGCCTAGAAA–(**5–1.2**)–CTGCAGTATTC CCGGGTTCATTAGAATTCCTT
***3′UTR*** **, “insulator context”**	**TAA**TCTAGAAAACAAACAAA–(**5–1.2**)–AAAAAGAAAAATAAAAAGAATTCCTT
***3′UTR*** **, “E3 context”**	**TAA**TCTAGAGCGCGTCGACAAAAAAAAAAGA–(**5–1.2**)–TGTGCTAAATAAACTA CAAATAAAAAGAATTCCTT
***3′UTR*** **, “distant context”**	**TAA**TCTAGATTCATTTTCTTTCATTTTCATTCGACACGCCGCGGCATCTTCGTTTTCTTCACCGATTAATTTTAAT–(**5–1.2**)–GAATTCCTT
***5′UTR*** **, “stem”**	AATTATCTAgagcaggagactgctcAACACAAAACTCGAGAACAT*ATG*

The start and stop codons for vYFPΔ are denoted by *ATG* and **TAA**, respectively. The beginning of the PGK terminator is underlined (CTT). The lower case text denotes the stem region in the *5′UTR*, “stem” context.

To understand the factors limiting use of a single *3′UTR* to achieve efficient vYFPΔ_**5–1.2** localization, we performed 3*′*–RACE on these transcripts. These experiments established that in all the sequence contexts used, polyadenylation occurred within the terminator region downstream of the aptamer. Sequencing data for the transcript containing the aptamer within the MS2 sequence context also confirmed that an intact aptamer with correct sequence was present, so a mutated or absent aptamer element does not explain the lack of localization. Next, we tested whether the **5–1.2** aptamer bound TetR with significantly lower affinity when presented within an RNA substrate simulating the *5′UTR* of the **5–1.2**_vYFPΔ transcript versus the *3′UTR* of the vYFPΔ_**5–1.2**. We observed similarly high affinity binding between TetR and **5–1.2** located in either the 5*′UTR* (K_d_ = 0.3 nM) or *3′UTR* (K_d_ = 0.8 nM) contexts (Fig. 5C and Table 2). These dissociation constants were similar to those previously measured for the isolated aptamer [24], [25]. Notably, no binding was observed in the absence of a functional TetR aptamer (Fig. 5C). Altogether, these data indicate that while a single intact aptamer is being encoded within the *3′UTR in vivo* and within sequence contexts that can mediate high affinity binding to TetR, RNA localization is significantly less efficient in comparison with what is achieved using a single aptamer within the *5′UTR*.

**Table 2:** Sequences of the *in vitro* transcribed RNA used in binding experiments.

Transcript	Sequence
*5′UTR* +aptamer	GGGAAUUAUCUAGGAUCCAGGCAGAGAAAGGUCGAUACGGACGGAAUGUGAUGGCCUGGAUCCAACACAAAACUCGAGAACAU*AUGUCU*AAAGGUGAAGAAUUAUUCACUGGUGUUGUCCCAAUUUUGGUUGAAUUAGAUGGUGAUGUUAAUGGUCACAAAUUUUCUGUCUCCGGUGAAGGUG
*3′UTR* +aptamer	GGGCUUGUUACCAGACAACCAUUACUUAUCCUAUCAAUCUGCCUUAUCCAAAGAUCCAAACGAAAAGAGAGACCACAUGGUCUUGUUAGAAUUUGUUACU**UAA**UCUAGAAAUUAUCUAGGAUCCAGGCAGAGAAAGGUCGAUACGGACGGAAUGUGAUGGCCUGGAUCCAACACAAAACUCGAGAACAUCCCGGGAAAAUCUAGAAUUCCUUCGAUAGAUCAAUUUUUUUCUUUUCUCUUUCCCCAUCCUUUACGCUAAAAUAAUAGUUUAUUUUAUUUUUUGAAUAUUUUUUAUUUAUAUACGUAUAUAUAGACUAUUAUUUAUCUUUUAAUGAUU
*5′UTR* no aptamer	GGGAAUUAUCUACUUAAGAACACAAAACUCGAGAACAU*AUGUCU*AAAGGUGAAGAAUUAUUCACUGGUGUUGUCCCAAUUUUGGUUGAAUUAGAUGGUGAUGUUAAUGGUCACAAAUUUUCUGUCUCCGGUGAAGGUG

The vYFPΔ start and stop codons are denoted by italicized *AUG* and bold **UAA**, respectively. The **5–1.2** aptamer is underlined.

Previous reports have indicated the importance of translational repression during transport for efficient mRNA localization [32], a scenario that is inherently achieved when our aptamer is located within the *5′UTR* [Fig. 2C and [25]]. Therefore, we tested whether using a highly structured *5′UTR* RNA element that can decrease the efficiency with which vYFPΔ_**5–1.2** is translated would improve its localization. This approach can be used to rescue proper asymmetric targeting of *ASH1* transcripts containing a single *3′UTR* localization element [37], [38]. We encoded a hairpin element previously shown to reduce translation rates in yeast [37] within the *5′UTR* of vYFPΔ_**5–1.2**. However, this modification alone was insufficient to confer efficient localization via a single *3′UTR* TetR aptamer (Fig. 5D).

### Transcript Localization using TetR Aptamer Arrays within the *3′UTR*

**Figure 6:**
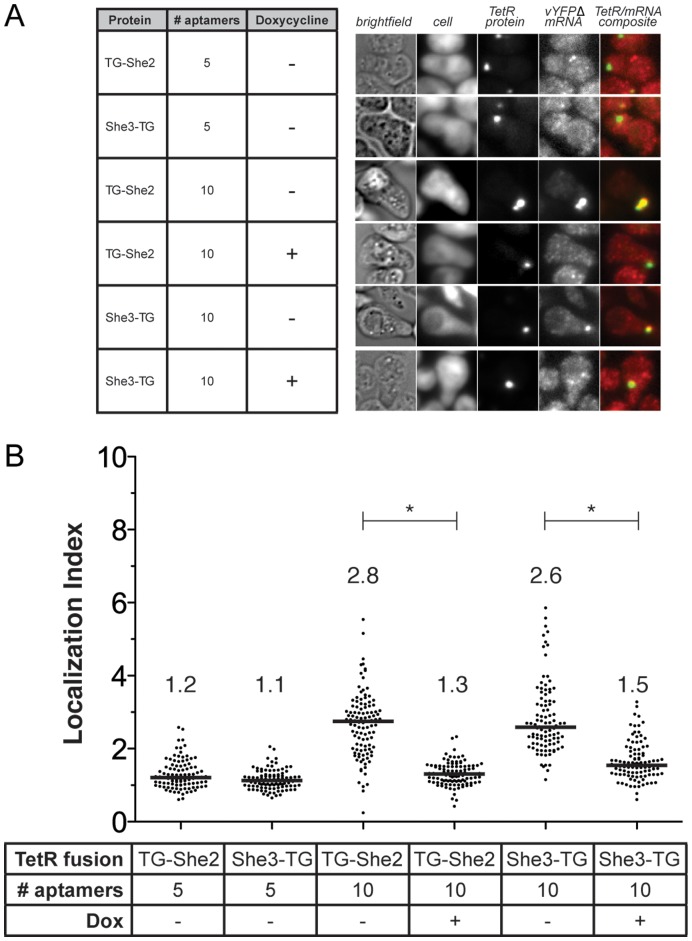
Within the 3′UTR, arrays containing a minimum of ten 5–1.2 aptamers are required to achieve transcript localization comparable to that observed for a single aptamer located within the 5′UTR. (A) Microscopy images of G1-phase arrested cells. The cell body, TG–She2 or She3–TG, and reporter transcript (vYFPΔ–(**5–1.2**)*_n_* (where *n* = 5 or 10) are visualized for cells co–expressing the indicated protein and reporter transcript. The absence or presence of doxycycline in the growth media is noted. (B) Localization Indexes determined for the indicated conditions are summarized. In all cases, the line and numbers above each data set represent the median Localization Index for that condition. A bar with an asterisk (*) above denotes a statistically significant difference (*p*<0.0001), as determined by a two-tailed *t*–test, between the indicated conditions.

Previous studies using the MS2 coat and λN RNA-binding proteins empirically demonstrated that increasing the number of potential protein binding sites within the *3′UTR* is necessary to demonstrate an effect by the tethered protein fusion [21], [39], [40], [41]. We created the vYFPΔ_(**5–1.2**)_5_ and vYFPΔ_(**5–1.2**)_10_ reporter constructs containing five and ten aptamer sequences within the *3′UTR*, respectively. No enhanced localization was observed using the vYFPΔ_(**5–1.2**)_5_ transcript with either TG–She2 or She3–TG (Fig. 6A and B). However, efficient and inducible localization of the vYFPΔ_(**5–1.2**)_10_ transcript with both She2–TG and She3–TG was achieved (Fig. 6A and B). The Localization Index determined using the vYFPΔ_(**5–1.2**)_10_ transcript closely matched that for the **5–1.2**_vYFPΔ (single aptamer in the *5′UTR*) transcript (Fig. 4B). The biochemical basis for requiring multiple *3′UTR cis*-elements to achieve efficient transcript localization is unclear. However, this phenomenon is not restricted to engineered transcripts, as several naturally localized and translationally regulated transcripts also contain multiple *3′UTR cis*-elements. Models [reviewed in [3]] proposing multivalent interactions in *trans* between *3′UTR cis*-elements or local clustering of RBPs to enhance recruitment of weakly interacting but essential components of the localization machinery have been put forward. However, the importance of these or alternative mechanisms to the TetR–aptamer and the various natural systems remain to be fully elucidated.

In summary, we have demonstrated an inducible system for directly controlling subcellular RNA localization. At the level of the target transcript, a single TetR aptamer positioned within the *5′UTR* in a context that also conferred inducible translational regulation by this system promoted effective transcript localization. In the *3′UTR*, however, at least ten tandem TetR aptamers were required to achieve localization efficiencies equivalent to those for a single aptamer placed in the *5′UTR*. At the protein level, TetR could be successfully fused with two different proteins encoding the necessary biochemical information to interface target transcripts ‘marked’ with TetR aptamers with the cellular RNA localization machinery. This was achieved without adverse impact on either TetR or its fusion partner’s function. We have not explicitly examined whether transcripts marked with TetR aptamers are trafficked within their own RNP complexes or co-packaged with natively targeted transcripts. However, in previous work, Lange *et al* showed that native transcripts trafficked in a She2p/She3p/Myo4p-dependent manner are co-packaged [42]. Therefore, it is reasonable to infer that transcripts tagged with TetR aptamers are co-transported with native transcripts.

Overall, our findings indicate the potential broader utility of the TetR–aptamer system in systematically dissecting the functional contribution(s) of individual RBPs and putative components of the RNA localization machinery to establishing the transcript–specific subcellular mRNA distribution patterns observed naturally. Indeed, it is estimated that between 2–8% of the genome of model eukaryotes encode RBPs, and these may act combinatorially and/or hierarchically to control various RNA fates, including localization [43]. Many of these are likely capable of sequence–specific RNA recognition [44] and, hence, may be potential players in the subcellular trafficking of specific mRNAs. In principle, simultaneously obtaining information on the subcellular co–localization of a candidate TetR–RBP fusion, its naturally associated RNA(s) and synthetic TetR–aptamer containing target transcripts could help elucidate whether a given RBP plays a dominant and indispensable role in proper transcript trafficking. Here, the ability to directly toggle the TetR–RBP interaction with the synthetic transcript serves as an important strategy for precisely confirming the biochemical basis for RNA localization, thereby minimizing confounding artifacts due to non–specific protein–RNA interactions. In a more applied context, our system also provides new opportunities for engineering precise and temporal control over RNA localization in areas such as neurobiology and developmental biology, where this process is of clear biological importance and potentially manipulated to gain fundamental knowledge and for biomedical applications.

## Materials and Methods

### General

Firefly luciferase levels were determined using a standard luciferase assay as described previously [25]. *In vitro* transcription and equilibrium binding experiments were performed using a cytometric bead binding assay [24]. Mapping of the *3′* ends of the reporter transcript was performed using *3′*–RACE [45]. All plasmids created during this work and reported within have been uploaded to GenBank and are summarized in Table 3.

**Table 3:** Summary of the plasmids used in this study.

Plasmid name	Plasmid backbone	Genbank Accession	Reference	Usage notes
**pRS–TG**	pRS416	JX679612	This work	microscopy, translation repression assay
**pRS–TG–She2**	pRS416	JX679613	This work	microscopy, translation repression assay
**pRS–She3–TG**	pRS416	JX679611	This work	microscopy, translation repression assay
**5–1.2–FLuciferase**	YCplac22	JX679607	Ref. 25	translation repression assay
**YCplac22**	YCplac22		Ref. 31	plate assay
**She2 [K5547]**	YCplac22		Ref. 31	plate assay
**YCp–TG–She2**	YCplac22	JX679622	This work	plate assay
**She3 [K4822]**	YCplac22		Ref. 31	plate assay
**YCp–She3–TG**	YCplac22	JX679621	This work	plate assay
**5–1.2–vYFPΔ**	pRS304	JX679608	This work	microscopy
**ADH1pro–5–1.2–vYFPΔ**	pRS304	JX679609	This work	microscopy
**No aptamer–vYFPΔ**	pRS304	JX679610	This work	microscopy
**vYFPΔ–5–1.2(5′)**	pRS304	JX679615	This work	microscopy
**vYFPΔ–5–1.2(MS2)**	pRS304	JX679620	This work	microscopy
**vYFPΔ–5–1.2(insulator)**	pRS304	JX679619	This work	microscopy
**vYFPΔ–5–1.2(distant)**	pRS304	JX679618	This work	microscopy
**stem–vYFPΔ–5–1.2**	pRS304	JX679614	This work	microscopy
**vYFPΔ–5–1.2(5x)**	pRS304	JX679616	This work	microscopy
**vYFPΔ–5–1.2(10x)**	pRS304	JX679617	This work	microscopy

### Plate-based Growth Assay

The K5547 (to test She2) or K4822 (to test She3) yeast strains harboring the indicated vector(s) was grown to saturation at 30°C in Synthetic Defined Media #1 (SD1) (6.7 g/L YNB, 20 mg/L adenine, 20 mg/L uracil, 100 mg/L leucine, 20 mg/L histidine) +20 g/L glucose. Cultures were diluted 10,000-fold into minimal media and grown for 16 hours. Yeast cultures were diluted serially ten-fold, and 5 µL of each dilution was spotted onto agar plates as indicated and grown at 30°C for three days before visualization. Tryptophan dropout plates contained SD1, 20 g/L agar and 20 g/L glucose. Plates lacking tryptophan and adenine contained SD1 without adenine, 20 g/L agar and 20 g/L glucose.

### Immunoblotting

Cells expressing the indicated proteins were grown to mid-log phase. Lysates were prepared by pelleting the yeast in a microcentrifuge at 5,000×*g* for 2 minutes. Cells were washed once in Spheroplast Buffer (50 mM Tris-HCl, pH 7.5, 1.4 M sorbitol, 40 mM *ß*–mercaptoethanol), and incubated at 37°C for 15 minutes in 100 µL Spheroplast Buffer containing 2 U zymolyase (Zymo Research) and protease inhibitors (Sigma). Laemmli sample buffer was added to a final concentration of 1× and samples were incubated for 10 minutes at 95°C. Samples were centrifuged at 12,000×*g* for 1 minute. The supernatants were loaded and separated by SDS–PAGE and transferred to a PVDF membrane. TetR was detected using an anti–TetR monoclonal antibody (Clontech, Clone 9G9).

### Fluorescence *in situ* Hybridization

The W303–1A yeast strains transformed with the indicated plasmids were grown to saturation at 30°C in Synthetic Defined Media #2 (SD2) (6.7 g/L YNB, 20 mg/L adenine, 100 mg/L leucine, 20 mg/L histidine) + 20 g/L glucose. Cultures were diluted 150-fold into 10 mL of SD2+20 g/L galactose to induce TetR-fusion expression, and grown for 16 hours at 30°C to mid-log phase. Alpha-factor mating pheromone (Zymo Research) was added to a final concentration of 10 µg/mL, and cultures were incubated at 30°C for four hours. Cells were fixed using formaldehyde (4% final concentration) and incubated for 40 minutes at room temperature. Cells were pelleted by centrifugation at 3,000×*g* and washed three times in cold Buffer B (BB) (1.2 M sorbitol, 0.1 M K_2_HPO_4_, pH = 7.5). Cells were resuspended in 1 mL BB containing 2U Zymolyase (Zymo Research) and 2 mM vanadyl ribonucleoside complex, and incubated for 15 minutes at 30°C. Cells were centrifuged at 850×*g*, washed twice in cold BB and incubated in 70% ethanol at room temperature for one hour. Upon harvesting by centrifugation at 400×*g*, cells were resuspended in 1 mL Washing Buffer (WB) (10% formamide, 300 mM NaCl, 30 mM sodium citrate, pH = 7.0), and incubated at room temperature for 3 minutes. Cells were pelleted by centrifugation at 400×*g* and resuspended in 100 µL Hybridization Buffer (10% dextran sulfate, 1 mg/mL yeast tRNA, 2 mM vanadyl ribonucleoside complex, 0.2 mg/mL bovine serum albumin, 10% formamide, 300 mM NaCl, 30 mM sodium citrate, pH = 7.0) containing 250 nM FISH hybridization probes, and incubated overnight. FISH probes consisted of a pool of 20–mer Cy5-labeled oligodeoxynucleotides specific for unique sequences within the target transcript (Biosearch). Cells were washed in 1 mL WB, collected by centrifugation at 400×*g* and resuspended in 1 mL WB and incubated at 30°C for thirty minutes. Cells were collected as above, resuspended in 100 µL of WB containing 1x Whole Cell Stain Blue (Thermo), and incubated at room temperature for thirty minutes. Cells were washed twice with 1 mL WB, resuspended in 2x SSC solution (300 mM NaCl, 30 mM sodium citrate, pH = 7.0), and spotted on glass slides for imaging.

### Microscopy and Image Analysis

Images of cells were captured using a Zeiss AxioObserver microscope with filters (Chroma) optimized for DAPI (Whole Cell Stain), GFP (TG, TG–She2 or She3–TG) and Cy5 (FISH probes) excitation and detection. Data acquisition was performed with Metamorph software (Molecular Devices). Automated image analysis was performed using the Cell Profiler software [46], [47]. Mask 1 surrounding the She2–TG and She3–TG foci were identified using the Otsu thresholding method on the GFP channel image, and all objects smaller than four pixels were discarded. The corresponding Mask 2 was detected by identifying secondary objects using the Watershed–Gradient and the Otsu thresholding methods on the DAPI–channel image, and clumped objects or objects on the edge of the image field were discarded. Background fluorescence was determined by measuring the Cy5–channel fluorescence of W303-1A yeast cells labeled with sequence scrambled FISH probes. The mean Cy5–channel fluorescence in Regions 1 and 2 were calculated. Cells with near or below background levels of signal in the Cy5 channel were excluded from further analysis. The Localization Index was determined on a cell–by–cell basis using the formula: mean fluorescence in Region 1/mean fluorescence in Region 2.
